# The temporary cost of dominance

**DOI:** 10.7554/eLife.68790

**Published:** 2021-04-30

**Authors:** Calen P Ryan, Christopher W Kuzawa

**Affiliations:** 1 Department of Anthropology, Northwestern UniversityEvanstonUnited States; 2 Institute for Policy Research, Northwestern UniversityEvanstonUnited States

**Keywords:** biological age, epigenetic clock, methylation, aging, *P. cynocephalus*

## Abstract

In a population of wild baboons, a new way to assess biological age reveals a surprising effect of social hierarchy.

**Related research article** Anderson JA, Johnston RA, Lea AJ, Campos FA, Voyles TN, Akinyi MY, Alberts SC, Archie EA, Tung J. 2021. High social status males experience accelerated epigenetic aging in wild baboons. *eLife* **10**:e66128. doi: 10.7554/eLife.66128

Aging is inevitable, but also unfair: individuals can share the same number of years, yet their bodies may be getting older at different rates. Accurate ways of measuring this ‘biological aging’ would help to assess which individuals in a population are aging faster, and why.

Epigenetic clocks measure the regular unfolding of chemical marks on DNA, and can predict chronological age – that is, the amount of time since birth (Horvath and Raj, 2018; Ryan, 2020). In many species, including humans, the difference between epigenetic and chronological age serves as an index for how quickly the body of an animal is aging. In fact, people who look epigenetically older than their chronological age tend to have shorter lives (Marioni et al., 2015). Now, in eLife, Jenny Tung and colleagues – including Jordan Anderson and Rachel Johnston as joint first authors – report that in a population of wild baboons from Amboseli National Park in Kenya, social factors might speed up epigenetic aging in unexpected ways (Anderson et al., 2021).

The team (who are based in various institutions in the United States and Kenya) developed an epigenetic clock that rivaled or exceeded better-established methods to estimate the chronological age of a baboon. Yet, despite this accuracy, some animals were predicted to be older or younger than their years. The Amboseli group has been closely observed for 50 years, and previous studies have shown that monkeys in this population had reduced lifespans if they faced challenges early in life (Alberts and Altmann, 2012). Such experiences included being born to a low-status mother, competing with siblings, and growing up during a drought or at times of high population density (Archie et al., 2014; Tung et al., 2016). In addition, having fewer social bonds has also been linked to reduced lifespans in this group (Silk et al., 2010). However, Anderson et al. found that none of these factors were associated with epigenetic aging.

In contrast, male baboons with higher dominance ranks – who fiercely compete for their place in the hierarchy – looked epigenetically older than their chronological age. This link was not seen in females, who do not fight for their social status but instead inherit it from their mother. To further explore the association between a male’s place in the social hierarchy and his biological aging, the researchers tested whether males who increased or dropped in rank also experienced changes in their epigenetic age (Figure 1). As predicted, males’ epigenetic clocks tended to accelerate with an increase in rank. Intriguingly, however, losing social status also made males look epigenetically younger. These findings suggest that the competitive behaviors which help males achieve dominance and increase mating opportunities also come at the price of biological wear and tear, and a sped-up epigenetic clock. However, these costs seem to be temporary and reversible.

**Figure 1.**
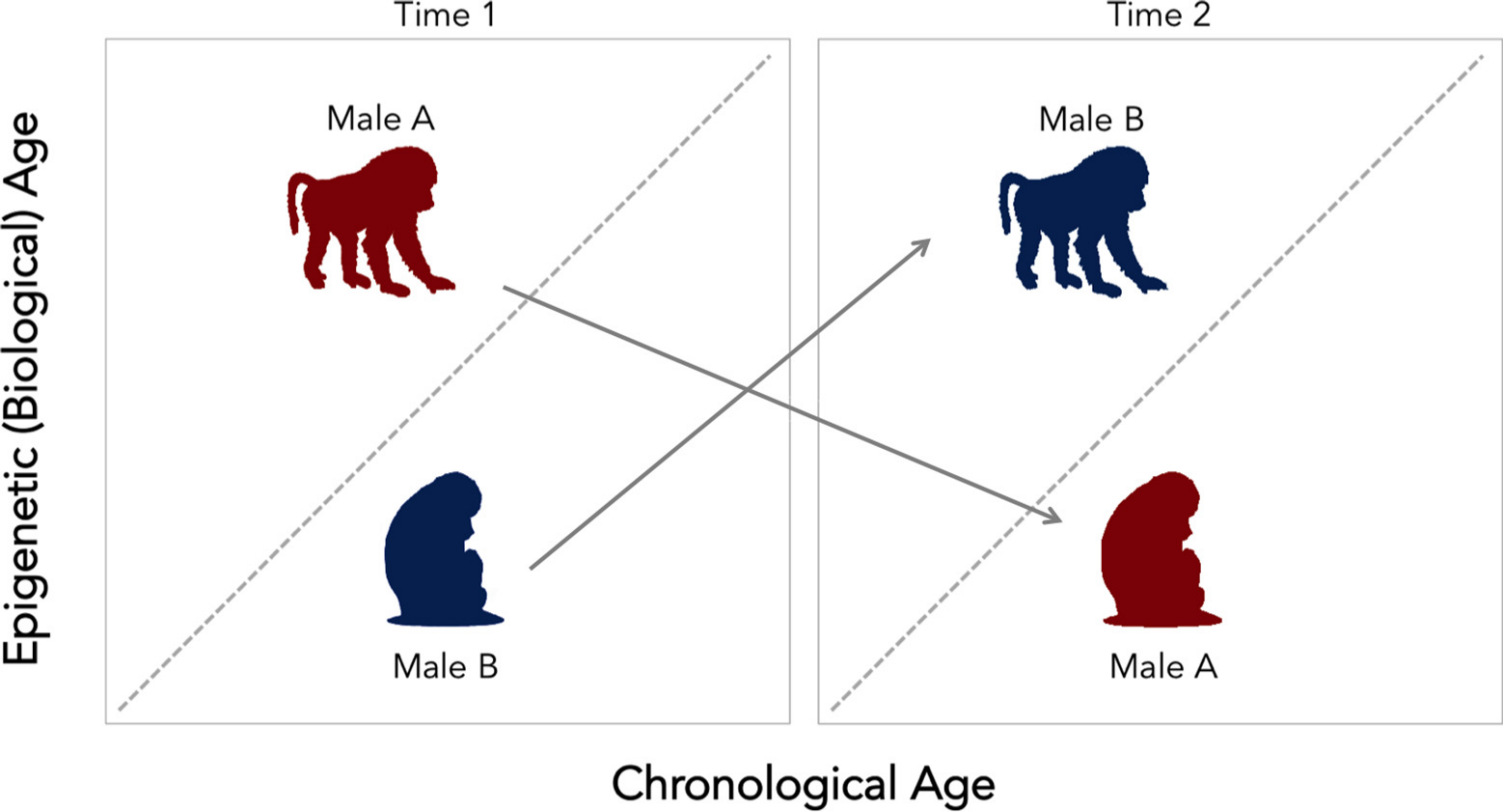
Change in social status alters the relationship between biological and chronological aging in wild baboons. Chronological age (how many years an individual has been alive for) can differ from epigenetic (that is, biological) age. The dotted diagonal line shows the rate where both measures match; individuals above this line are biologically older than their actual age, and individuals below are biologically younger than their chronological age. Male baboons who are socially dominant (upright, walking figure) tended to be epigenetically ‘older’ than expected given their chronological age. This relationship was reversed for male individuals of lesser ranks (sitting figure). However, changes in social status between two time points (gray arrows) alter the speed of the epigenetic clock. Low status males (male B, in blue) which gained dominance tended to become epigenetically ‘older’ relative to chronological age. In contrast, when a dominant male (male A, in red) lost status, his epigenetic age tended to decline. These findings suggest that the epigenetic clock accelerates as baboons gain social dominance, but that these aspects of biological aging are transient. This could mean that this epigenetic measure might not be associated with mortality or lifespan, as it is found for other species.

This study is among the first to develop an epigenetic clock to explore the social determinants of epigenetic aging in a wild animal population, and confirm that this method tracks chronological age quite well. Yet the findings by Anderson et al. also raise new questions about the dimensions of biological change that epigenetic clocks may capture. For instance, it is still unclear why dominance rank predicts the acceleration of epigenetic age in male baboons, while other factors associated with lifespan in both sexes do not. More generally, the biological meaning of this sped-up clock remains to be explored in this population. If males can appear older or younger than their chronological age based on their current dominance rank, how do these transient effects impact their lifespan or functional decline – as similar measures have been shown to do in other species (Marioni et al., 2015; Stubbs et al., 2017)?

The transient effect of dominance on epigenetic age was a surprising finding that will likely require more work to unpack. The epigenetic clock that Anderson et al. developed was based upon analysis of genetic material from immune cells present in the blood, and may therefore reflect changes in the immune profile of an individual at the time they were sampled. In fact, switches in dominance rank in male baboons are accompanied by shifts in immune function (Lea et al., 2018); this might explain how the clocks could temporarily reflect a male’s current status. Future research in wild animals like the Amboseli baboons, especially in the social and natural environments in which they evolved, will help further dissect epigenetic clocks, and clarify what makes them tick.
